# Specifying cross-system collaboration strategies for implementation: a multi-site qualitative study with child welfare and behavioral health organizations

**DOI:** 10.1186/s13012-024-01335-1

**Published:** 2024-02-12

**Authors:** Alicia C. Bunger, Emmeline Chuang, Amanda M. Girth, Kathryn E. Lancaster, Rebecca Smith, Rebecca J. Phillips, Jared Martin, Fawn Gadel, Tina Willauer, Marla J. Himmeger, Jennifer Millisor, Jen McClellan, Byron J. Powell, Lisa Saldana, Gregory A. Aarons

**Affiliations:** 1 Division of General Internal Medicine, Department of Internal Medicine, College of Medicine, The Ohio State University, Columbus, OH USA; 2 School of Social Welfare, University of California Berkeley, Berkeley, CA USA; 3 John Glenn College of Public Affairs, The Ohio State University, Columbus, OH USA; 4 School of Medicine, Wake Forest University, Winston-Salem, NC USA; 5 Sheps Center for Health Services Research, University of North Carolina at Chapel Hill, Chapel Hill, NC USA; 6 Social Science Division, Western Oregon University, Monmouth, OR USA; 7 Center for Vulnerable Populations, Department of Medicine, University of California, San Francisco, CA USA; 8 Public Children Services Association of Ohio, Columbus, OH USA; 9 Children and Family Futures, Irvine, CA USA; 10 Center for Mental Health Services Research, Brown School, Washington University in St. Louis, St. Louis, MO USA; 11 Center for Dissemination & Implementation, Institute for Public Health, Washington University in St. Louis, St. Louis, MO USA; 12 Division of Infectious Diseases, John T. Milliken Department of Medicine, School of Medicine, Washington University in St. Louis, St. Louis, MO USA; 13 Lighthouse Institute, Chestnut Health Systems, Eugene, OR USA; 14 Department of Psychiatry, University of California, San Diego, La Jolla, CA USA

**Keywords:** Collaboration, Implementation strategies, Cross-system interventions

## Abstract

**Background:**

Cross-system interventions that integrate health, behavioral health, and social services can improve client outcomes and expand community impact. Successful implementation of these interventions depends on the extent to which service partners can align frontline services and organizational operations. However, collaboration strategies linking multiple implementation contexts have received limited empirical attention. This study identifies, describes, and specifies multi-level collaboration strategies used during the implementation of Ohio Sobriety Treatment and Reducing Trauma (Ohio START), a cross-system intervention that integrates services across two systems (child welfare and evidence-based behavioral health services) for families that are affected by co-occurring child maltreatment and parental substance use disorders.

**Methods:**

In phase 1, we used a multi-site qualitative design with 17 counties that implemented Ohio START. Qualitative data were gathered from 104 staff from child welfare agencies, behavioral health treatment organizations, and regional behavioral health boards involved in implementation via 48 small group interviews about collaborative approaches to implementation. To examine cross-system collaboration strategies, qualitative data were analyzed using an iterative template approach and content analysis. In phase 2, a 16-member expert panel met to validate and specify the cross-system collaboration strategies identified in the interviews. The panel was comprised of key child welfare and behavioral health partners and scholars.

**Results:**

In phase 1, we identified seven cross-system collaboration strategies used for implementation. Three strategies were used to staff the program: (1) contract for expertise, (2) provide joint supervision, and (3) co-locate staff. Two strategies were used to promote service access: (4) referral protocols and (5) expedited access agreements. Two strategies were used to align case plans: (6) shared decision-making meetings, and (7) sharing data. In phase 2, expert panelists specified operational details of the cross-system collaboration strategies, and explained the processes by which strategies were perceived to improve implementation and service system outcomes.

**Conclusions:**

We identified a range of cross-system collaboration strategies that show promise for improving staffing, service access, and case planning. Leaders, supervisors, and frontline staff used these strategies during all phases of implementation. These findings lay the foundation for future experimental and quasi-experimental studies that test the effectiveness of cross-system collaboration strategies.

**Supplementary Information:**

The online version contains supplementary material available at 10.1186/s13012-024-01335-1.

Contributions to the literature

- Successful implementation of cross-system interventions requires cross-system collaboration strategies to improve implementation, service delivery, and client outcomes.
- We identified and specified seven cross-system collaboration strategies. Contracting out, joint supervision, and co-location were used to staff the program. Referral protocols and expedited access agreements were used to promote service access. Shared decision-making meetings and data sharing were used to align case planning.
- Cross-system collaboration strategies were used at the individual- and organizational-levels throughout the preparation, implementation, and sustainment phases.
- Our work on cross-system collaboration strategies advances the specification of functions and forms of bridging factors in implementation.

## Background

Individuals and families affected by substance use disorders (SUDs) and trauma have complex service needs that span health, behavioral health, and social service systems. Cross-system interventions that integrate services can improve client outcomes and expand community impact. These interventions include service cascades and clinical pathways that identify service needs in one system (e.g., child welfare) and link to specialized treatment in another (e.g., SUD treatment) to create a continuum of care [1, 2]. Cross-system interventions are challenging to implement because they involve multiple stakeholders and intervention components that must be aligned across different systems [3, 4]. Differences in the values, priorities, regulations, funding, staffing, and capacity in two systems can undermine collaboration [5] and delay or threaten the implementation and sustainment of cross-system interventions [6, 7].

*Cross-system collaboration strategies* address these contextual barriers by aligning operations and services across systems for implementation [8]. These strategies are essential for effective cross-system implementation and service delivery [9, 10]. However, like many strategies used for addressing system and organizational complexity, collaboration strategies have received insufficient empirical attention [11], which limits the scale-up, and public health impact of cross-system interventions. This study identified, described, and specified cross-system collaboration strategies used to implement Sobriety Treatment and Recovery Teams (START) in Ohio (USA) as Sobriety Treatment and Reducing Trauma (Ohio START). START is an evidence-based cross-system intervention linking child welfare and SUD treatment for parents.

### Cross-system collaboration strategies for implementation

Strong interorganizational collaboration is critical for implementing cross-system interventions [2, 10, 12–14]. For example, collaboration difficulties limited implementation in prior studies of cross-system interventions focused on screening, assessing, and referring children served by the child welfare system to community-based behavioral health services [7, 15–19]. Collaboration problems included contract disruptions, poor communication, limited familiarity with external providers and their services, unclear referral protocols, and disagreements about data sharing [7, 15–19]. Addressing these challenges is critical [20], but can be difficult and time-consuming [21–23] especially during early phases of preparation and implementation [24]. Relational implementation strategies that build or leverage relationships (e.g., network weaving, obtaining formal commitments, building a coalition) could guide collaborations for implementation [25, 26]. However, these strategies are limited in their specificity and few studies have examined how these strategies are used during implementation.

*Cross-system collaboration strategies* align operations and services across systems for implementation [8]. These strategies fall into a larger category of “bridging factors” linking the outer and inner contexts identified in the Exploration, Preparation, Implementation, Sustainment (EPIS) framework [27] and serve as the “connective tissue” [11] of organizations and systems. Drawing on theories on interorganizational relationships, cross-system collaboration strategies can serve several functions (purposes or goals). From an economic perspective, organizations may collaborate to access new and complementary resources to support their operations (resource dependence theory) [28] or create efficiencies (transaction cost economics) [29]. From an institutional theory perspective, organizations collaborate to respond to external pressures, coordinate services for their clients, or pool influence for policy and system planning [30, 31]. Additionally, the specific forms of collaboration (ways or methods) for accomplishing these functions can vary. Prior literature distinguishes between administrative and frontline collaboration [32]. *Administrative* forms of collaboration align organizational operations and include formal or informal agreements to contract for services, joint budgeting, co-locate or cross-train staff, share data, or develop joint programming [32–38]. Administrative-level partnerships support collaboration on the *frontline* where practitioners engage in a different set of collaboration strategies including making referrals or activities to align case plans [36, 39]. Yet, little is known about how these collaboration strategies are used, how they support implementation, or the conditions under which they are effective.

### Study purpose and context

The purpose of this study is to identify, describe, and specify cross-system collaboration strategies used to implement START, an evidence-based cross-system intervention linking child welfare and behavioral health systems in Ohio. Ohio START requires that county child welfare agencies develop formal partnerships with at least one local behavioral health organization. However, the specific forms and functions (ways and reasons why) of strategies that child welfare agencies use to collaborate with behavioral health providers during START implementation can differ. These distinctions may explain differences in implementation and effectiveness while informing implementation practice. This study offers a foundation for future trials that examine the effectiveness of collaboration strategies for improving the implementation, reach, and effectiveness of cross-system interventions.

## Methods

### Intervention and setting

Ohio START is an affiliate of the national Sobriety Treatment and Recovery Teams (START) model. The national START model has demonstrated evidence of effectiveness for improving parental SUD treatment access, sobriety, and family reunification [35, 40–45] based on prior quasi-experimental studies (using propensity score matched comparisons) and is listed in several best practice registries [46, 47]. Ohio START includes several sequenced practice components including SUD screening completed by child welfare workers (required in Ohio, but not the national model), shared decision-making meetings (SDMM) that bring child welfare, behavioral health, and other service professionals together with families to plan services; wraparound support from a family peer mentor (FPM) who has lived experience with recovery and child welfare involvement; and expedited access to SUD treatment within 38 days of the child welfare case opening. The parent study protocol provides more intervention details [8]. To date, Ohio START has been implemented in 53 Ohio counties; counties began implementing in cohorts beginning in 2017.

The state of Ohio provides an ideal setting for identifying a wide range of local collaboration strategies. With a strong home-rule tradition, public services are administered by local county governments—for example, across the 88 counties in the state, there are 85 county public child welfare agencies (3 counties consolidated), and 49 regional behavioral health boards that conduct local planning efforts. These counties range in population size from 1.3 million (Franklin County) to 12,565 (Vinton County) and span diverse geographic contexts (urban, suburban, rural, and Appalachian). This context is important because the SUD treatment availability, community needs, and collaborative histories that shape decisions about the functions and forms of collaboration vary across different county settings [30].

### Design

We used a two-phased approach as follows: phase 1 used a multi-site qualitative design [48] (*n* = 17 counties) that drew on qualitative data to identify and define specific cross-system collaboration strategies used to implement Ohio START. Phase 2 included an expert panel of community partners and scholars to validate, explain, and specify the cross-system collaboration strategies according to strategy specification guidelines [49].

### Phase 1: qualitative multi-site study

#### Site selection

Sites were defined as Ohio counties implementing Ohio START (which included the county child welfare agency and their behavioral health partners). We included all 32 county child welfare agencies that had implemented Ohio START for at least 1 year as of November 2019. Eligible agencies included 17 that began implementing in 2017 with cohort 1 and 15 that began implementing in 2018 with cohort 2. Directors from 17 county agencies (cohort 1, *n* = 9; cohort 2, *n* = 8) agreed to participate. Most counties (*n* = 11; 64.7%) were rural (no urban core), 6 were in the Appalachian region of the USA. Fewer counties were suburban (*n* = 2, 11.8%) or urban (*n* = 4, 23.5%). In our original protocol [8], we planned to recruit only cohort 1 counties since they had the most implementation experience, but expanded to counties from cohort 2 to increase the number and geographic diversity of sites.

#### Data collection

##### Participants and recruitment

We conducted 48 small group interviews [50] with 104 unique staff involved in Ohio START implementation across the 17 counties. Our objective was to conduct at least 2 small group interviews in each county (1 with child welfare staff and at least one with behavioral health staff). This amounted to 17 interviews with 52 child welfare staff (leaders, supervisors, and frontline workers) and 25 interviews with 44 behavioral health staff (supervisors and clinicians) (from 15 counties; behavioral health providers from two counties declined to participate) (Table 1). Small group interviews included both administrative and frontline key informants to identify cross-system collaboration strategies at multiple levels. Given the emerging role of regional behavioral health boards in supporting cross-system alignment during the cohort 2 roll-out, we also conducted interviews in 6 counties with eight board representatives. The research team sent an initial recruitment email to the main contact at each child welfare agency and asked them to identify other Ohio START staff for recruitment. During the interviews with child welfare agency team members, we identified the main behavioral health partners; afterwards, the research team sent recruitment emails to those individuals. Behavioral health board directors in cohort 2 counties were emailed recruitment invitations.

**Table 1:** Phase 1—site and participant characteristics (*n* = 17 counties)

	Site/counties (*n* = 17)	Participants (*n* = 104)
*Organizational representation*		
Child welfare	17	52
Substance use treatment	15	44
Behavioral health boards	6	8
*Cohort*		
1	9	55
2	8	9
*County type*		
Urban	4	23
Suburban	2	14
Rural	5	37
Appalachian	6	30
*County population size (based on all counties in state)*		
0–25th percentile (< 36,980)	3	14
26–50th percentile (36,981–58,552)	5	33
51–75th percentile (58,553–126,764)	3	19
76–100th percentile (+ 126,765)	6	38

##### Procedures

Prior to the interviews, all participants received the consent script and questions. Consent was obtained verbally at the beginning of each interview. Interviews were 60 min long and conducted via video conference and facilitated by at least two research team members. Research team members included master- or doctoral-level investigators with training in qualitative methods and familiarity with the Ohio START program. The semi-structured interview guide prompted for general impressions about collaboration and implementation of the Ohio START program in each county, partnerships between child welfare and SUD treatment organizations, and working relationships between behavioral health boards (Supplementary file 1). For interviews conducted after COVID-19 mitigation closures began, participants were also asked about pandemic effects on collaboration and implementation of Ohio START. Interviews were recorded and professionally transcribed; team members also completed debrief summaries after interviews to guide initial codebook development. Participants were offered a $30 gift card.

##### Analysis

We used a template approach drawing on constructs from our conceptual model (published elsewhere [51]) and themes that emerged from our interview debrief notes to develop an initial codebook. A focal set of codes was generated to reflect cross-system collaboration strategies reported in interviews. Two team members independently applied the codebook to each transcript and refined the codebook iteratively (Supplementary file 2). We validated our 7 cross-system collaboration strategies with our expert panel. The research team discussed discrepancies to reach a shared understanding and consistent application. We extracted the content of the strategy codes to determine the strategies used in each county and generate general descriptions consistent with strategy reporting guidelines [49]. With these descriptions, we organized the 7 strategies into 3 categories based on their function (staff the program, promote service access, align case plans).

### Phase 2: expert panel

We used a participatory process to further specify each cross-system collaboration strategy consistent with system-strategy development methods [52–54]. We convened a 16-member expert panel that was purposely selected to reflect diverse salient experiences and expertise. The panel included 6 community partners (3 child welfare representatives, the START purveyor, 2 behavioral health leaders) who had firsthand experience implementing START in Ohio, 3 experts in implementation science with experience operationalizing implementation strategies in child welfare systems outside of Ohio, and 7 research team members (doctoral-level scholars and trainees) who collected and analyzed the phase I qualitative data. All of our panelists collaborated together on the study design via virtual meetings, document review, and asynchronous communication. The expert panel was convened to integrate panelists’ experience and knowledge to (1) check and validate initial phase 1 findings about the 7 collaboration strategies with community partners and (2) specify the strategies of each strategy by building on phase 1 findings about collaboration strategies used in START implementation and (3) refine our specification of each collaboration strategy (i.e., definitions, mechanisms for action, and outcomes).

Originally, we planned in-person meetings for our collaborative design process; however, due to the COVID-19 pandemic, we instead met during five 1–2 h virtual sessions to specify strategies. To prepare, we developed a strategy specification spreadsheet that outlined operational details for each of the identified cross-system collaboration strategies based on available interview data which was shared with panelists ahead of time. During our meetings, we focused on one strategy at a time, clarified definitions, and refined specification details. Panelists were prompted to specify the causal logic for how each strategy supported START implementation and the specific implementation and service system outcomes targeted. Afterwards, the research team refined and finalized our cross-system collaboration strategies (Supplementary file 3). We also shared these strategies and their definitions with a group of Ohio START implementation technical assistance providers (implementation support professionals) to verify face validity and to identify other collaboration strategies they observed but were not reflected in our interview data, or expert panel discussions.

## Results

**Fig. 1:**
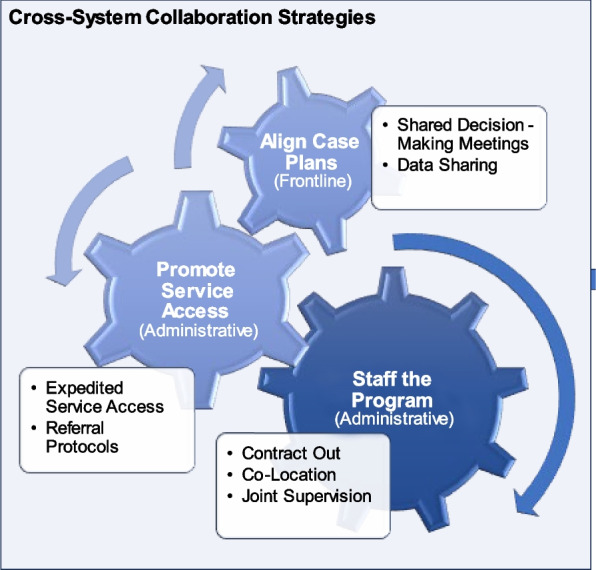
Cross-system collaboration strategies for implementation

**Fig. 2:**
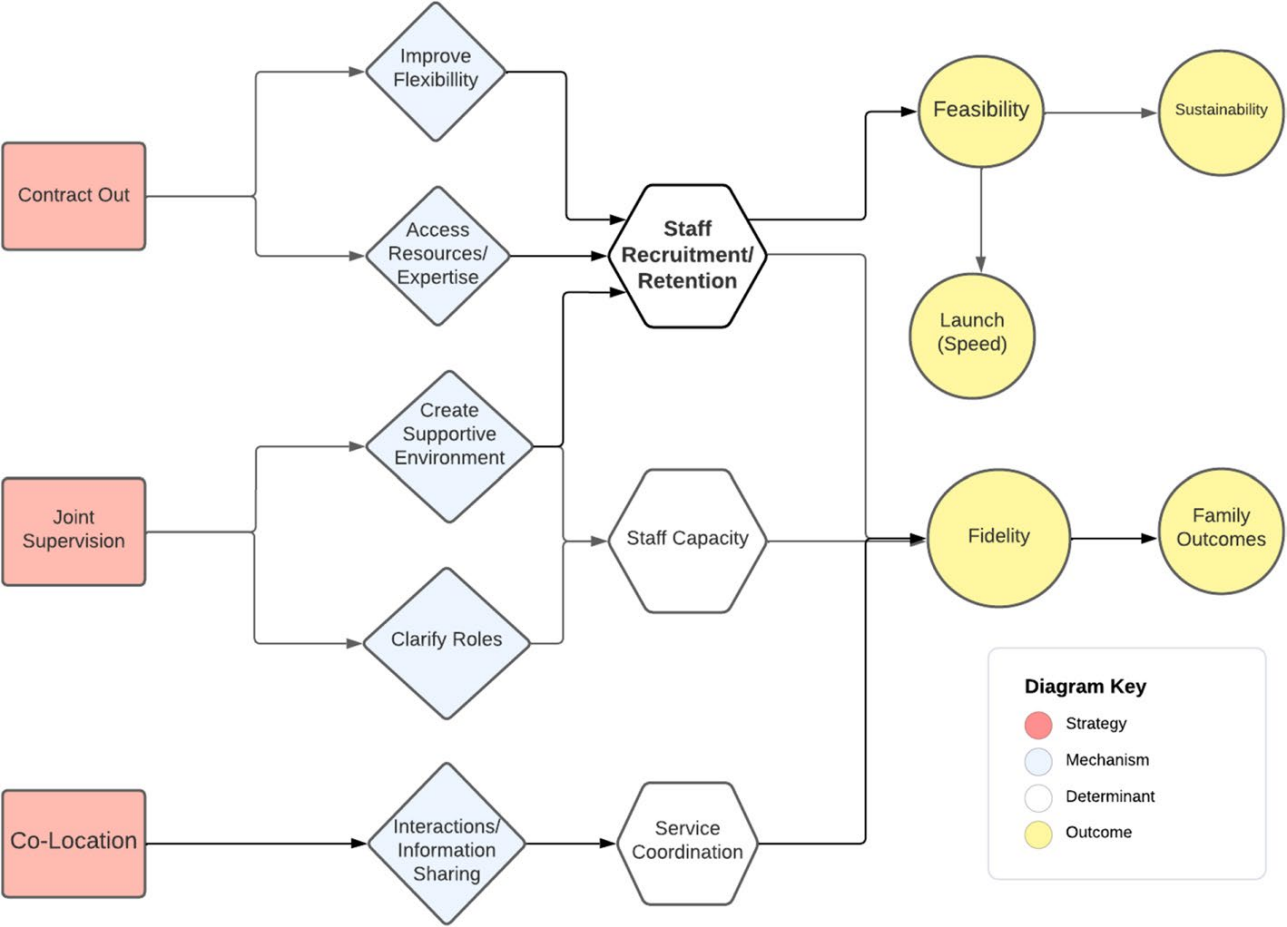
Cross-system collaboration strategies to staff the program

**Fig. 3:**
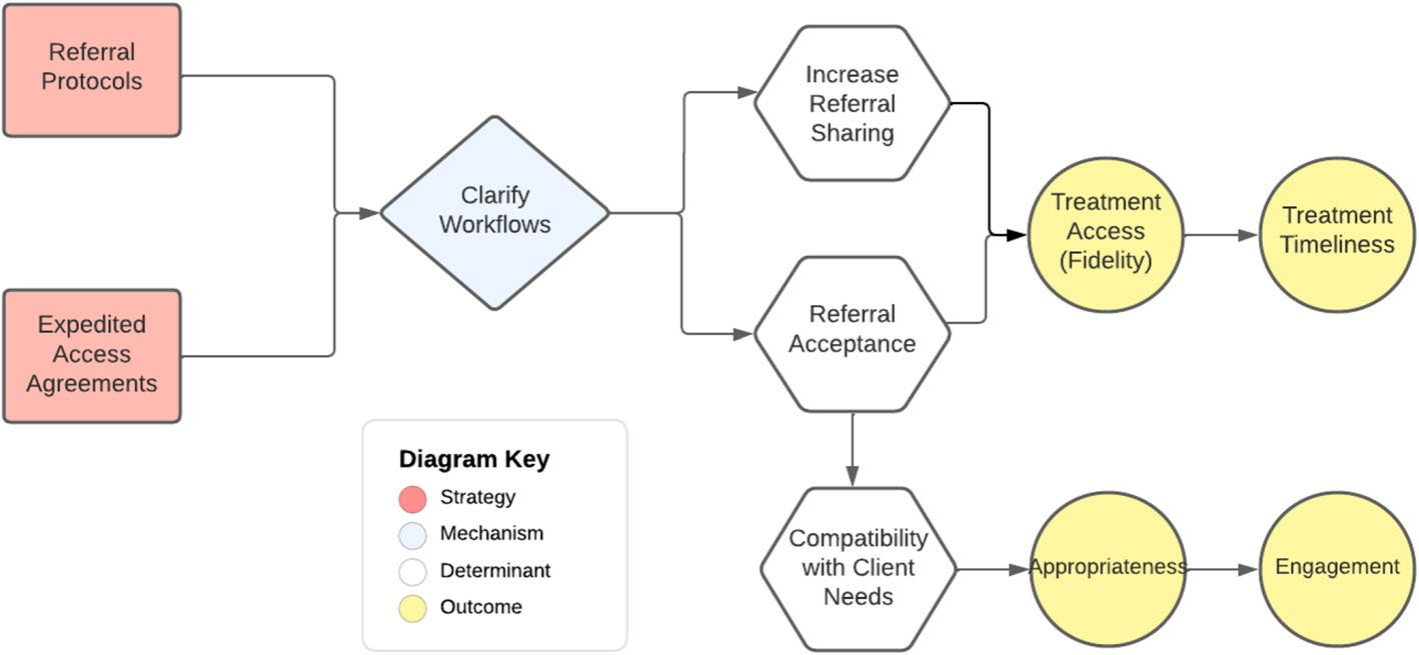
Cross-system collaboration strategies to promote service access

**Fig. 4:**
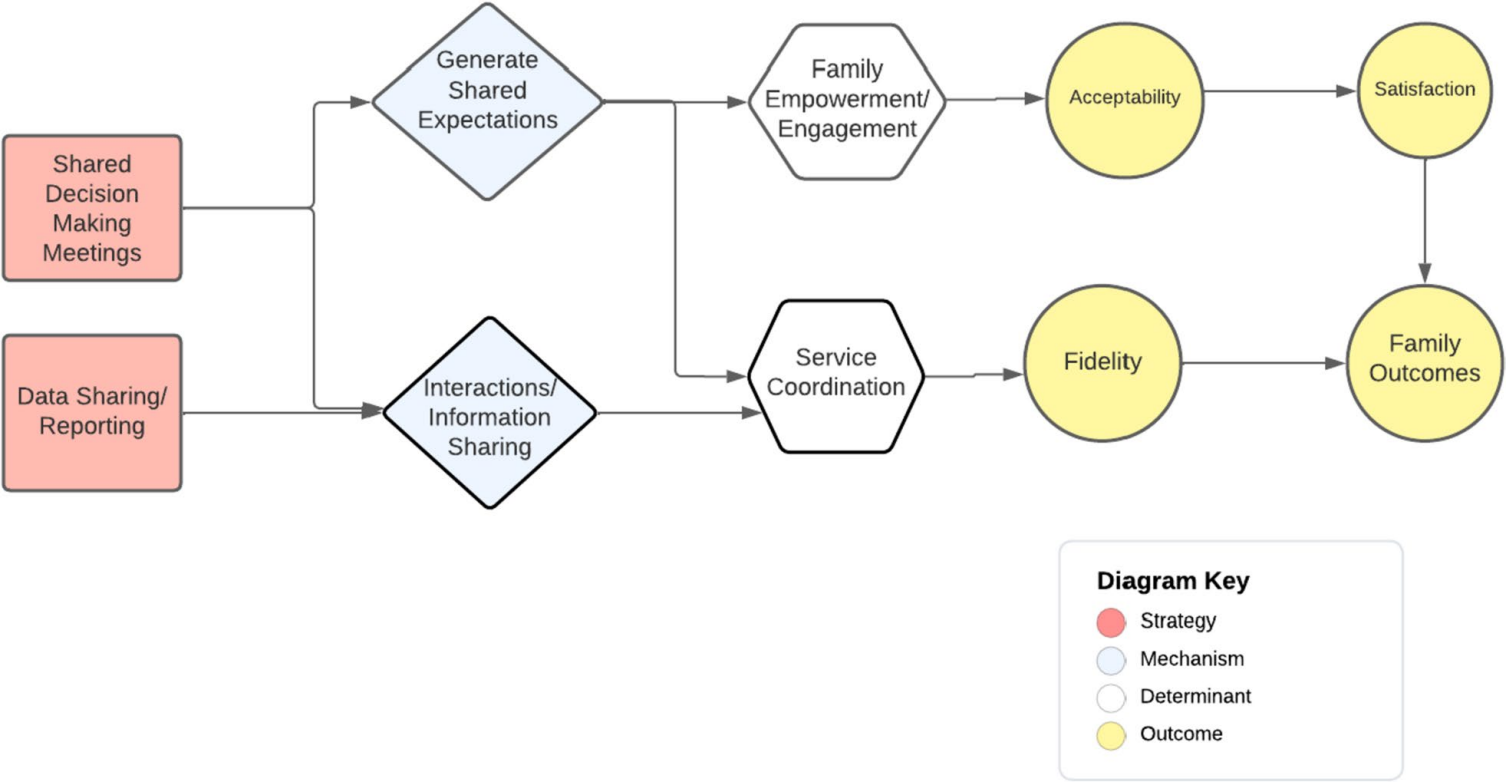
Cross-system collaboration strategies to algin case plans

We identified seven collaboration strategies that collectively accomplished three functions in relation to Ohio START implementation: staffing the program, promoting service access, and aligning case plans. These strategies were used at different levels of the organization (e.g., administrative, frontline) and were intended to improve implementation and service system outcomes (Table 2, Fig. 1). Below, we define and describe each strategy and provide an example application within Ohio START based on case study results. Then, we draw on expert panel discussions to describe hypothesized mechanisms of action and targeted outcomes; mechanisms are also diagrammed using a casual pathway diagramming approach (Figs. 2, 3, and 4) [55, 56].

**Table 2:** Cross-system collaboration strategies—definitions, mechanisms, and outcomes as specified by the expert panel

	**Strategy/form & definition**	**Mechanisms**	**Determinants addressed**	**Implementation outcomes**	**Service outcomes**
**Function: staff the program (administrative collaboration)** Actors: agency leaders (including human resource, procurement professionals), supervisors					
1	*Contract out*: Contracting out for expertise involves outsourcing a staff role needed to implement a particular program/model to another organization. This entails an agreement that the staff person in this position is employed by another organization for purposes of supporting the EBP/program in the focal organization	Improve flexibility Access new resources	Staff recruitment/retention Staff capacity	Feasibility Speed (launch) Fidelity (overall) Sustainability	–
2	*Joint supervision*: Supervision for a staff person is delivered by individuals from more than one organization. This supervision might be delivered at the same time or separately; specific types of supervision might be split across organizational supervisors	Supportive environment Clarify roles			
3	*Co-location*: Employees from a partner organization work within another organization and are provided the same organizational resources/supports as other employees (e.g., desk, building access) to facilitate intentional interaction, and communication among staff within and across organizations. Co-location is considered the foundation that helps move toward more seamless coordination and integration	Promote interactions	Service coordination	Fidelity (overall)	–
**Function: promote service access (administrative collaboration)** Actors: agency leaders, supervisors					
4	*Referral protocols*: Supervisors and other agency leaders develop and carry out agreed-upon procedures for referring clients for services at another external organization. There may or may not be a formal written agreement between the two organizations	Clarify workflows	Referrals Compatibility	Fidelity (access) Appropriateness	Timeliness (access) Engagement
5	*Expedited access agreements*: An explicit and formal agreement between two organizations to provide services to one another’s' clients to implement a new model/program in a particular way, for a specified price/term, and/or other conditions				
**Function: align case plans (frontline collaboration)** Actors: supervisors, front-line clinicians, and caseworkers (with support from agency leaders)					
6	*Shared decision-making meetings*: Joint meetings of all caseworkers, clinicians, staff, peer specialists, family members, and family supporters to discuss the case goals, progress, and plans for a family consistent with a new program/model. These meetings are intended to set objectives and align services for a family	Shared expectations Promote interactions	Service coordination Family engagement	Fidelity (overall) Acceptability	Patient centeredness
7	*Data sharing*: Exchanging information about client case plans, service needs, progress, and completion to implement the new program/model. This can take multiple forms including formal reports shared regularly with partners, inputting data and using a shared data system intended for sharing case files, or more ad hoc information sharing about cases	Promote interactions

### Strategies for staffing the program

Three collaboration strategies were focused on staffing: contract out for expertise, co-location, and co-supervision (Fig. 2). Staffing strategies were typically used by top-level administrators (including human resource and contract procurement professionals) and supervisors.

#### Contract out for expertise

Interview participants described how agencies often contracted out specific staff roles. This often involved a formal agreement (contract or memorandum of understanding [MOU]) with a partnering organization to hire or designate an existing employee to staff the program in the focal agency. Fourteen Ohio START counties contracted with a behavioral health partner to staff the FPM role, an essential intervention component but not a typical position in public child welfare agencies. Agency leaders and procurement professionals negotiated and executed these agreements during early planning and preparation but revisited and adjusted during active implementation.

It worked out really well when we were recruiting a FPM … [the behavioral health organization] provides peer mentoring services across multiple counties, and it was helpful … because they already knew who was engaged in the recovery community and who wasn’t. -Child welfare agency representative

Child welfare representatives explained that contracting with a partner organization supported implementation in two ways. First, interview participants described how contracting out was perceived as a more flexible approach to staffing than hiring in-house. Contracting out allowed county agencies to work around hiring restrictions and lengthy recruitment processes associated with hiring public employees, which could delay program launch. Second, expert panel participants explained that contracting out also allowed child welfare agencies to access behavioral health organizations’ connections with peer support communities and expertise around hiring, supervising, and supporting FPMs. This specialized expertise and connection were anticipated to improve the recruitment and retention of strong FPMs who implement the model with fidelity. Expert panelists also noted that behavioral health providers can bill the state Medicaid program for peer recovery support services while public child welfare agencies rarely do, thus accessing new financial resources to sustain the program. Together, these benefits were perceived to improve feasibility.

#### Joint supervision

Interview participants described joint supervision, which is delivered by individuals from more than one organization to staff to ensure that staff have access to appropriate institutional knowledge and skills needed to effectively perform their work. Supervisors from different organizations might overlap or differ in specific types of supervision provided (administrative, clinical, supportive), and this might occur simultaneously or at different stages during implementation. In Ohio START, 13 counties reported using (*n* = 10) or an intention to use (*n* = 3) joint supervision of FPMs by staff from each partnering organization. Joint supervision typically entailed administrative and clinical supervision of co-located FPMs by child welfare agency representatives and supportive supervision by behavioral health supervisors (focusing on self-care, recovery, peer ethics, etc.). The assignment of multiple supervisors allowed for a more extensive support system to help FPMs navigate child welfare and behavioral health systems during implementation. Participants described the potential for conflicting supervisory objectives and priorities and the need for supervisors to coordinate.

The point and purpose of having the co-supervision is so the peer mentors and the staff members working closely with the agencies have a better understanding of the standards and procedures and the practice of child welfare, [which] works very differently than [behavioral health partner] and vice versa. So, it’s again, just to bring everybody up to a common understanding. -Behavioral health provider

Expert panelists described how joint supervision can lead to strong implementation by providing a supportive environment for FPMs and clarifying their roles which might be important for FPMs in boundary-spanning roles. By improving the job support available to FPMs, panelists expected that joint supervision would increase FPM capacity, and retention, which would support implementation and sustainment with fidelity.

#### Co-locate staff

Interviewees reported on staff co-location, where employees from a partner organization were physically co-located within another agency and provided designated workspace, equipment, and resources. In 12 Ohio START counties, FPMs hired by partnering behavioral health organizations were physically co-located with child welfare agency staff. Participants described how co-location brings FPMs and child welfare workers into physical proximity and facilitates communication and service coordination compared to other counties where child welfare workers and FPMs worked in different settings. Notably, representatives from several counties noted that space limitations and remote work during the first year of the COVID-19 pandemic prevented co-location which required FPMs and caseworkers to be more planful about their communications.

Our FPMs are co-located in our offices with the child welfare team. They spend more time here than they do in their technical employer’s office. … We do a lot of joint meetings and supervision. I think that has gone very well. -Child welfare agency representative

Expert panelists posited that co-location influences implementation by facilitating more frequent interactions and streamlining communication among staff from different organizations. Through interactions, panelists expected that staff would build trust, skills for resolving conflicts, and strong working relationships needed for effective service coordination. Because well-coordinated services are essential for Ohio START, co-location was expected to support overall fidelity (Fig. 2).

### Strategies for promoting service access

Two collaboration strategies emerged for promoting service access: referral protocols and expedited access agreements (Fig. 3). Agency leaders and supervisors used these strategies.

#### Referral protocols

Interviewees described developing and using referral protocols which are agreed-upon procedures for referring clients for services. Some of these agreements were formalized in contracts or MOUs, while others were informal. In Ohio START, six counties reported how representatives from child welfare agencies and substance use treatment provider partners developed referral protocols. These protocols specified criteria for referring parents for particular types of treatment (e.g., mothers with custody of young children who need inpatient treatment), information shared by the child welfare worker regarding the parent and their case, the SUD treatment agency point of contact who should receive the referral/information, and procedures for following up with the original referring caseworker to confirm that the referral has been received. Referral protocols were often developed during preparation and revisited during active implementation.

The referral process is more streamlined with START. It’s really helped to improve coordination in terms of this is somebody who’s being sent as part of the START program, the understanding is there that the weekly reports will be sent out at that point, and it just makes the process much more streamlined.

-Behavioral health provider

Expert panelists explained that referral protocols influenced implementation by clarifying workflows across systems because they reflect a solidified and shared understanding of how organizations will share and receive referrals. Developing referral protocols was intended to provide a clear workflow for frontline workers making and accepting referrals that can improve the likelihood of treatment delivery (fidelity) and expedite service access (timeliness). Panelists also noted how clarifying the nature of services available and eligibility criteria also enhances the likelihood of linking parents to the most appropriate services for their needs, thereby improving engagement in care.

#### Expedited access agreements

Participants reported developing expedited access agreements which were typically codified in a contract or MOU and specified how each partner would provide services to a client group within a given time frame. Expedited service access agreements were negotiated and executed among agency leadership during implementation preparation but were described as playing an important role in facilitating implementation. In Ohio START, four counties had expedited access agreements. Interviewees described how these agreements were used to ensure that START-enrolled families received behavioral health services within the recommended timeline (four treatment sessions within 38 days of case opening to child welfare). When present, expedited access agreements allowed parents in Ohio START to “skip the line” in communities with long waitlists for SUD treatment, therefore improving the speed of treatment delivery. Service timeliness is a critical component of the START model because parents’ ability to demonstrate progress toward sobriety within 12 months of their children entering foster care affects reunification outcomes. Behavioral health providers also described expedited access agreements as benefitting their agencies due to reduced client no-shows.

We signed the contract … in which we would prioritize [child welfare] referrals and get assessments done within a certain timeframe. They were struggling with some of their current providers in the area having long waiting lists. And so, they reached out to us.

-Behavioral health provider

Expert panelists explained how formal expedited access agreements influenced implementation. Institutionalizing shared understanding of how partnering organizations will work together and on what time frame was expected to lead to timely service delivery. Having timely treatment available “on demand” at the time when a parent was ready to engage in treatment was also perceived to enhance the compatibility and appropriateness of the model. Formal agreements were described as more robust in the event of staff or other institutional change, therefore enhancing sustainment.

### Strategies for aligning case plans

Two cross-system collaboration strategies aligned case plans: shared decision-making meetings (SDMMs) and data sharing (Fig. 4). Caseworkers, clinicians, and other frontline professionals used these strategies with support from supervisors and top-level administrators.

#### Shared decision-making meetings (SDMMs)

According to interviewees, SDMMs included all frontline providers and family members involved in a case coming together to collectively set goals, review progress, and align service plans across organizations. SDMMs took place during active implementation and sustainment, and ideally with top-level leader support. In this study, all counties reported using SDMMs to align case plans every 90 days and upon significant case events (e.g., reunification, treatment transitions, case closure). Meetings included caseworkers, behavioral health clinicians, staff, peer specialists, family members, and family supporters. Most meetings occurred in person, and behavioral health clinician attendance was challenging in some counties because productivity expectations and service demand precluded them from using their time for non-billable activities. Interviewees noted the potential for phone or virtual participation, and seeking alternative funding to ensure behavioral health clinicians are engaged.

I feel like SDMMs help bring everybody that’s involved with the family together and make sure that they’re all on the same page. If they have any questions, they can be answered… it also gives them the chance to have a voice and say what they feel and need to say. … So far, it’s been going great. I think it’s super helpful in helping the case move forward. -Child welfare agency representative

Expert panel participants explained that SDMMs supported implementation by promoting interactions among frontline professionals and family members, which facilitated information sharing, shared understanding of the situation, and clear expectations to improve service coordination and fidelity to the model. Because SDMMs were intended to center the family in service decisions, expert panelists anticipated that SDMMs empowered families, potentially enhancing model acceptability and patient-centeredness.

#### Sharing data/case-level information

Interview participants, particularly frontline caseworkers and clinicians described data sharing as the exchange of information about individual clients’ case plans, service needs, progress, and completion. Data sharing occurred during active implementation and sustainment in multiple ways including formal reports shared with partners, inputting data and using a shared data system, or more ad hoc information sharing about cases (e.g., phone calls). In Ohio START, all counties shared case information. Most child welfare agencies (*n* = 14, 82%) specified the expected data type or format in a contract or MOU and expected weekly communication. Child welfare and behavioral health professionals described tension and barriers to case-level information sharing due to different preferences and needs in relation to their roles in working with children and their families. For example, behavioral health providers preferred receiving information that would help them individualize treatment for their client, the parent(s) (e.g., concerns that motivated the referral, screening results). Child welfare workers preferred information that indicated a safety risk for children so that they could make appropriate recommendations about parental reunification or custody arrangements (e.g., parent attendance at treatment sessions, results of drug testing). Child welfare staff from several counties felt frustrated that behavioral health providers did not provide adequate or timely information about parents and their progress in treatment. Behavioral health providers in these counties reported that child welfare partners often probed for more details about families than they felt comfortable sharing and therefore “guarded” information to preserve confidentiality and trust with their client. This “guarding” of information often led parents to perceive that behavioral health workers were “on their side” and child welfare workers were not, which is inconsistent with the START model’s team approach. In counties that reported satisfactory information sharing, participants noted the importance of early and honest discussion among frontline partners during preparatory phases and arriving at a mutually satisfactory agreement about the type of information that will be shared, frequency, data protections, and purpose.

I keep open contact and communication with my providers even if I’m having a face-to-face during the week and something’s just not normal with my family. I would send an email directly to the provider so that the provider could talk to that family regarding that type of behavior. I also send assessment tools that I use in my interviews with Ohio START to my providers so that they can also look out the same lens that I’m looking out of, so that we can all stay on the same page. -Child welfare agency representative

Expert panelists described how sharing case-level information leads to strong implementation because case workers and clinicians align their knowledge about a family through their interactions. This information should trigger a response by child welfare and behavioral health providers to coordinate and adjust services rapidly (and between SDMMs) based on evolving family needs so that the family can remain intact. Regularly shared information was expected to lead to more coordinated services for families, which could potentially enhance acceptability and fidelity.

## Discussion

Implementing cross-system interventions like Ohio START requires strong inter-organizational collaboration. However, research on collaboration has not kept pace with practice with limited empirical attention to describing, specifying, and testing collaboration strategies. Through our 17-county multi-site qualitative study and expert panel, participants identified and specified seven collaboration strategies that facilitated implementation. Consistent with earlier thinking on interorganizational relationships, these collaboration strategies were used to align organizational operations, programming, and frontline service delivery [36, 57]. The expert panel identified plausible mechanisms through which each strategy could improve implementation and service system outcomes. This study responds to calls for more thorough reporting of bridging factors like collaboration strategies by specifying functions and forms [11]. Our results also generated new insights about hypothesized mechanisms of action that could be tested in future studies examining the impact of cross-system collaboration strategies for implementing cross-system interventions that bridge siloed health and human service delivery systems.

### Staffing strategies align administrative resources to improve feasibility and fidelity

Administrative cross-system collaboration strategies to staff the program (contracting out for expertise, joint supervision, co-location) aligned child welfare and behavioral health human resources for implementation. These strategies were used by top-level administrators to address key barriers to implementation related to specialized staffing capacity and service coordination. Participants noted that in public agencies, contracting out often allowed for swifter program implementation than hiring in-house and allowed for more flexible expansion of agency capacity; in Ohio START, contracting also allowed staffing costs to be shifted to the behavioral health system (who could seek reimbursement from public and private health payers), which made the program more feasible and sustainable. Notably, agencies that contracted out for staff also tended to use co-location and joint supervision, suggesting that these three strategies might be bundled especially when implementing at the intersection of public and private systems. A transaction cost economics perspective might be useful for framing further studies examining how leaders evaluate the costs of contracting out versus hiring in-house and the conditions under which these staffing options enhance feasibility [58, 59]. Although participants in this study suggest that contracting out for staff at private organizations might be more flexible and quicker than hiring within public organizations, we still do not know if one strategy is better than the other for implementing interventions with fidelity or sustaining interventions long-term. It might be the case that job demands and resources for a position differ across public and private organizations which has implications for staff turnover and implementation success. It is also possible that agencies change their staffing approach in later implementation phases (e.g., an agency might contract-out for a family peer mentor initially to launch a program, but later create an in-house position to sustain it). These collaborative staffing decisions and impacts on implementation fidelity, feasibility, and sustainment are an important avenue for future research.

### Service access strategies align referral pathways for fidelity and timeliness

Cross-system collaboration strategies to promote service access (referral protocols and expedited access agreements) aligned organizational procedures across systems reflecting a blend of administrative and frontline functions. Supervisors and leaders used these collaboration strategies to align referral pathways, but frontline professionals (caseworkers and clinicians) were ultimately responsible for executing referrals. This suggests that formal agreements alone are insufficient for collaborative implementation and the importance of communicating the contents of formal agreements with frontline professionals [37].

Expedited access agreements and referral protocols were perceived to clarify workflows across organizations and treatment timelines in response to referral barriers, and concerns about service timeliness. These service access strategies were perceived to improve fidelity, appropriateness, and timeliness of service delivery—therefore, they might be especially important when implementing interventions that address time-sensitive needs, when there is limited time for intervention (e.g., emergencies, brief hospitalization), or to leverage client motivation to change [60]. Future studies are needed to test these and other potential strategies to determine their impact on implementation speed and service timeliness [61] considering the inconsistent evidence about whether referral protocols lead to quicker treatment [62]. Additional research is needed to investigate the ethics and potential unintended consequences [63] of expedited access agreements. Although these agreements can facilitate quick treatment access [60], they might also privilege clients based on their case status (e.g., status as a parent/guardian) or those referred from one type of agency over another regardless of treatment needs (e.g., [64]) which has potential to create or widen inequities in child welfare outcomes. Expedited or priority agreements could also preference providers that can accommodate new clients immediately, which tend to be private and for-profit [65], or overemphasize timely access at the expense of the client’s choice of provider [66].

### Case planning strategies align frontline services for fidelity and acceptability

Finally, cross-system collaboration strategies to align case plans (through SDMMs and data sharing) reflected frontline collaboration. These strategies fostered interactions, information sharing, and shared expectations among frontline professionals to improve service coordination and family engagement. To promote case plan alignment across systems, frontline professionals engaged in extensive information and data sharing in meetings; expectations were specified in formal partnership agreements. While clear protocols and expectations are important, effective case alignment probably also depends on strong communication and interpersonal skills [67]. As our findings about the tensions that arise around sharing information about families suggest, clear and direct conversations that set shared goals, address different expectations across systems (e.g., the nature of information that can be shared), and resolve conflicts are likely to be important during preparation, implementation, and sustainment phases. It should also be noted that front-line staff might be reluctant to share information because of governmental rules and regulations designed to protect client privacy and confidentiality [67]. It might be useful for regulatory agencies to provide clear guidance to staff in both systems about how to comply with these rules without compromising collaboration.

Case alignment strategies focused on improving frontline service coordination for individual families by promoting interactions and information sharing among frontline professionals so that the model can be implemented with fidelity. SDMMs were expected to enhance intervention acceptability, client-centeredness, and parent satisfaction. Our findings that SDMMS were expected to generate shared goals and expectations with families, promote full engagement, and clinical success align with prior literature on shared decision-making practices [68, 69]. Strategies to align services likely require intensive coordination, adjustment, and frequent calibration of services [70]. Future studies are needed to examine the forms, frequency, and types of participants needed for effective case-planning collaboration during implementation, as well as strategies for addressing common barriers (e.g., different goals or policies that impede information sharing).

### Combining cross-system collaboration strategies at multiple levels

Our results highlight the many ways that organizations collaborate across systems throughout implementation. These strategies were used to initially launch the intervention and continue operations. In all 17 counties, participants described combining administrative and frontline cross-system collaboration strategies. These intensive partnerships based on multiple forms of collaboration are considered multiplex and have been linked to better performance and outcomes [71, 72]. As suggested in our expert panel discussions, administrative forms of collaboration (strategies for staffing and promoting service access) during preparation lay the foundation for strong frontline collaboration (strategies for aligning cases) during active implementation (continued operation) and sustainment. Our panelists’ hypotheses are supported by prior studies—for instance, co-location and MOUs are associated with greater service coordination and referrals to treatment [60, 73, 74]. In other sites where START has been implemented, organizations used administrative collaboration strategies to problem-solve, which provided the support and infrastructure frontline clinicians needed later to collaborate and implement START with fidelity [75, 76]. When considered along with evidence about the importance of early preparation activities for later implementation success [77], our findings imply that implementers should plan for collaboration strategies and potential collaboration challenges with their partners before program launch to support later implementation and sustainment of cross-system interventions. Future studies are needed to test hypotheses about the sequencing or additive effects of multiplex collaborative relationships on implementation, service delivery, and client outcomes.

While participants reported using similar forms of frontline collaboration, there was variation in administrative-level collaboration. Administrative-level collaboration involves sharing critical organizational resources (e.g., funding, physical space, staff expertise) for which organizations might also be competing with one another [32, 38, 78]. As a result, administrative-level collaboration strategies might be riskier and used less frequently than frontline collaboration (e.g., referrals, case planning, data sharing) [38, 57, 79, 80]. Considering how collaboration at multiple levels seems to be common for implementing cross-system interventions in our study, and important for effective service delivery [57, 72], attention to supporting administrative collaboration might be needed. This has implications for implementation facilitators, technical assistance providers, and other implementation support professionals who might focus on building trust and communication among organizational leaders as they negotiate and execute their partnerships [81, 82]. Given the use of formal agreements like contracts and MOUs, engaging procurement professionals and the frontline professionals who enact them is also critical for implementation [37].

### Limitations and future directions

Although our study drew from diverse county contexts and participants, it is likely that we did not identify all cross-system collaboration strategies for implementing cross-system interventions. For instance, our community partners acknowledged how other strategies like steering/planning committees and staff cross-training might also support implementation. Given the study timing (during COVID-19), our findings might overemphasize collaboration forms that do not require in-person contact. Future studies that examine the costs, time, and effectiveness of different collaboration formats (virtual, in-person, mediated by technology, etc.) have the potential to identify efficient approaches to cross-system collaboration and implementation. Similarly, continued research is needed to examine and specify functions and forms of collaboration strategies for implementation in different settings. The majority of our sites were smaller rural counties where there is a history of collaboration among systems and organizational leaders; therefore, our findings might overemphasize the frequency and intensity of collaboration. It bears noting that Ohio’s strong county-administered structure provides for local rule, which might make it easier for government agencies to collaborate intensively with other local organizations (compared to agencies at the state or national level with larger bureaucracies). Also, there was strong external support for Ohio START from state government which might have led to more local collaboration for implementation in this study than in settings where governmental enthusiasm for implementation is more limited.

Notably, our study does not evaluate the effectiveness of cross-system collaboration strategies although our expert panel results offer hypotheses for future trials. Consistent with a contingency theory approach that posits that the most effective organizational structures and processes depend on the context [59], we expect that collaboration strategies that work well in one county might not be similarly effective in another. Therefore, additional research is necessary to examine the conditions under which cross-system collaboration strategies are most effective to uncover moderators.

The rich description and specification of cross-system collaboration strategies identified here lay the foundation for future studies that test their effectiveness for implementing cross-system interventions with the potential to improve service access and outcomes. For instance, these strategies are also likely to be applicable to implementing other types of cross-system interventions such as multisystemic therapy [83], intensive wrap-around care [84], and clinical pathways [85]. The contributions of the expert panel increased the robustness of our research findings and improved the usability/applicability of the cross-system collaboration strategies for practitioners and scholars. The hypothesized mechanisms of action generated from our expert panel explained how different cross-system collaboration strategies might be targeted to specific implementation challenges that affect implementation and service system outcomes, and respond to calls for more theory-building work in implementation [86].

## Conclusion

This study identified and specified seven collaboration strategies. These strategies were used to align administrative operations and frontline services across systems during implementation. Future studies are needed to test the effectiveness of these strategies for implementing cross-system interventions and improving families’ service access. Our work has the potential to support cross-system intervention implementation by helping leaders identify collaboration strategies that address implementation challenges.

### Supplementary Information

**Additional file 1. Interview guide. **

**Additional file 2.** Collaboration strategies codebook.

**Additional file 3.** Strategy specification grid view.

## Data Availability

De-identified data will be made available from the first author upon reasonable request.

## Abbreviations

COVID-19: Coronavirus disease 2019
FPM: Family peer mentor
MOU: Memorandum of understanding
Ohio START: Ohio Sobriety Treatment and Reducing Trauma
SDMM: Shared decision-making meeting
START: Sobriety Treatment and Recovery Teams
SUD: Substance use disorder
