# Supplementary material for: Specifying cross-system collaboration strategies for implementation: a multi-site qualitative study with child welfare and behavioral health organizations
Source: Implement Sci. 2024 Feb 12;19:13. doi: 10.1186/s13012-024-01335-1 (PMC10863233; doi:10.1186/s13012-024-01335-1)
Supplement: Supplementary file 1 — Additional file 1. Interview guide. [file 13012_2024_1335_MOESM1_ESM.docx]

**Aligning Behavioral Health & Child Welfare Systems to Address the Opiate Crisis in Rural Ohio** (RWJF)

**Collaborating to Implement Cross-System Interventions in Child Welfare and Substance Use** (NIDA)

Interview Topics for Child Welfare Leaders – (updated 02.27.20)

1. Warmup
   1. Can you tell me a little bit about your role in your PCSA and how long you've been in that role?
   2. In your own words, tell me a little about START in your county.
   3. Thinking about relationships between child welfare and behavioral health systems in your county, what’s working well?
2. Substance Use Treatment - Let’s first focus on relationships with substance use treatment providers.
   1. Strategy – How are you collaborating with behavioral health providers to implement START?
   2. Rationale – Tell me about how this relationship came to be…
   3. Strengths & Challenges – What are some of the strengths and challenges of your relationship
3. ADAMH – Now we are going to shift gears and talk about how your agency and your ADAMH board are connecting around Ohio START.
   1. History – How have your PCSA and your ADAMH board worked together in the past?
   2. Ohio START - How are you and your ADAMH board connecting to implement START?
   3. Strengths/Challenges – What are some of the strengths and challenges that have come up in your relationship with your ADAMH board?
4. Wrap Up
   1. If you could return to the beginning, what would have done differently with your relationships?
   2. What advice would you give other PCSA leaders?
   3. Is there anything else you’d like to tell us that we didn’t ask about?
5. Demographics – We will wrap up with a few quick demographic questions, that we would like to ask for reporting purposes back to our funders. (You are welcome to share this information with me privately via email if you prefer, or can decline to respond).
   1. How would you describe your gender identity?
   2. How would you describe your race/ethnicity?
   3. What is your disciplinary background?
