# Supplementary material for: Specifying cross-system collaboration strategies for implementation: a multi-site qualitative study with child welfare and behavioral health organizations
Source: Implement Sci. 2024 Feb 12;19:13. doi: 10.1186/s13012-024-01335-1 (PMC10863233; doi:10.1186/s13012-024-01335-1)
Supplement: Supplementary file 2 — Additional file 2. Collaboration strategies codebook. [file 13012_2024_1335_MOESM2_ESM.docx]

Supplementary File. Collaboration Strategies Codebook

| **Code** | **Definition/example** |
| --- | --- |
| E1. Contracts | **Presence or absence of contract or MOU.** "We have an MOU and contract… They're expired, but we have them…. [When setting it up] we had a lot of mutual conversation about nuts and bolts of salary and benefits… monthly progress reports… " |
| E2. Use of family peer mentors | **Perceptions of family peer mentor role or what the role entails.** |
|  | **Supervision of family peer mentors.** "There's a joint supervision model… Our behavioral health counterpart supervises them weekly. For Children's Services, it's been a crap shoot. Who is available at the time… The caseworker tends to get the more case-specific questions." |
|  | **Difficulty recruiting or retaining for the position.** "We've had some family peer mentor turnover which essentially puts everything on hold as far as new referrals and bringing new people in…" |
| E3. Data sharing | **Policies and procedures related to data sharing.** E.g., "We spend a lot of time talking with [behavioral health provider] about 42 CFSR Part 2… I hear three times a week about release of information…" OR "Interviewer: Is there a universal release or universal documentation that is the same for all the different START team members? Respondent: Yes... FAIR and JFS share a release of information as well..." |
|  | **Frequency and type of formal data (e.g., written reports/documentation) shared or not shared.** e.g., "Never have I worked with a substance use provider… where they wouldn't release full diagnostic assessments... full ISPs or treatment plan... Everything is very superficial… Attendance report -- show or didn't show. Positive or negative drug screen..." OR "We have a progress note that all provider agencies put into a shared system... so [with START], instead of submitting monthly, provider agencies should be submitting that weekly if it's a START client." |
| E4. Policies and procedures | Agency expectations and rules for collaboration and communication. |
| E5. Communication processes | **Formal and informal meetings and other communication processes (e.g., telephone, email) NOT already captured in E3 (data sharing) or E6 (FTMs)**. |
| E6. Family team meetings | Shared Decision Making Meetings (sometimes also referred to as Family Team Meetings). |
| E7. Other strategies | **Co-location.** "Our family peer mentors are co-located in our offices with the child welfare team. They spend more time here than they do in their technical employer's office." [Note: This would be double-coded with E2 "Use of family peer mentors."] |
